# Gene therapy within our vision: Illustrating the genetic paradigm

**DOI:** 10.4103/0974-620X.57307

**Published:** 2009

**Authors:** Sandy Raeburn

**Affiliations:** Clinical Genetics, University of Nottingham, UK

Ophthalmology was one of the first medical specialties to be studied by genetic methods, which were described in a classic textbook.[1] The importance of the eye in genetics was emphasized further, when, in 1962, Arnold Sorsby became the first editor of the Journal of Medical Genetics.[2] The review of Gene Therapy in this issue, from the Narayana Nethralaya Postgraduate Institute of Ophthalmology in Bangalore, is timely.[3] It is useful to consider how specific gene therapy fits into the context of services for the diagnosis and management of genetic disorders,[4] with a focus on eye disease.

We could summarize three lessons: Firstly, investigation should start with precise history-taking (in the patient and family), which is followed by careful clinical examination (affected people as well as those apparently unaffected). Clinical testing and more specialized techniques such as imaging or DNA studies should always come later. This sequence is the only validated approach to genetic diagnosis, apart from screening in populations that are at higher risk. If specific therapy is anticipated, this paradigm is essential. Modern clinicians must not be tempted to take short cuts, e.g. by studying DNA in patients and relatives before the clinical features and the family history have been delineated in detail and have been recorded. Searching for some mutations without a clinical lead will generate confusing data and usually negative results. Unless there is a lucky selection, or a particular mutation is frequent in a population, such molecular 'fishing' will not clarify the genetic cause. The precise clinical diagnosis facilitates identification of the causative gene. The accurate family history shows up clinical heterogeneity and clarifies the mode of inheritance.

The second lesson is that symptoms and signs in other organs may underpin the correct diagnosis. Hospital services need clinical equipment for testing even more now than they did before the genetic revolution; the clinical physiology of all affected people, e.g., the nerve conduction velocity or the electroretinogram (ERG), will help to discriminate between some closely related syndromes.

Thirdly, other treatments must be considered, before resorting to gene therapy. This is because for the foreseeable future more visual problems will be prevented or treated globally by such methods, than by gene therapy.

Ophthalmologists can study rare disorders of the eye effectively by following the diagnostic approaches above and collaborating with molecular genetic researchers with special interests in the causative genes. The paradigm above is as robust in research as in clinical practice. Ophthalmologists will often encounter conditions, which are genetically heterogeneous. The interest there in helping the patient and family is great, but, in addition, the research rewards are excellent.

The most logical model for gene therapy is in treating single gene disorders. Good ophthalmology practice will reveal many more treatable single gene conditions like Leber′s congenital amaurosis, which is the advance guard for other gene therapies.[5,6]
